# Splenic responses play an important role in remote ischemic preconditioning-mediated neuroprotection against stroke

**DOI:** 10.1186/s12974-018-1190-9

**Published:** 2018-05-28

**Authors:** Chen Chen, Wei Jiang, Zongjian Liu, Fengwu Li, Jian Yang, Yanlong Zhao, Yuanyuan Ran, Yan Meng, Xunming Ji, Xiaokun Geng, Huishan Du, Xiaoming Hu

**Affiliations:** 1 China-America Institute of Neuroscience, Beijing Luhe Hospital, Capital Medical University, Beijing, 101100 China; 2 Department of Pathology and Pathophysiology, School of Basic Medical Sciences, Capital Medical University, Beijing, China; 3 Institute of Hypoxia Medicine, Xuanwu Hospital, Capital Medical University, Beijing, China

**Keywords:** Cerebral ischemia, Spleen, Lymphocytes, Limb remote ischemic preconditioning

## Abstract

**Background:**

Remote ischemic preconditioning (RIPC) of a limb has been reported to protect against ischemic stroke. Our previous results demonstrated that the RIPC-mediated neuroprotection is associated with alterations in circulating immune cell populations. Here, we evaluated the effect of the spleen, the largest reservoir of immune cells, on RIPC-mediated neuroprotection against stroke.

**Methods:**

Noninvasive RIPC was achieved by four repeated cycles of 5-min blood flow constriction in the hindlimbs using a tourniquet. The blood and spleens were collected before and 1 h and 3 days after preconditioning to analyze the effect of RIPC on the spleen and the correlation between splenic and peripheral lymphocytes. Moreover, spleen weight and splenic lymphocytes were compared in stroke rats with or without RIPC. Finally, splenectomy was made 1 day or 2 weeks before RIPC and 90-min middle cerebral artery occlusion (MCAO). The infarct areas and deficits were assessed. Blood was collected 1 h after RIPC and 3 days after MCAO to explore the impact of splenectomy on RIPC-induced neuroprotection and immune changes. The contralateral and ipsilateral hemispheres were collected 3 days after MCAO to detect the infiltration of immune cells after RIPC and splenectomy.

**Results:**

Flow cytometry analysis demonstrated that the RIPC promptly increased the percentages of CD3^+^CD8^+^ cytotoxic T (Tc) cells in the spleen with a relatively delayed elevation in CD3^+^CD161^+^ natural killer T (NKT) and CD3^−^CD45RA^+^ B lymphocytes. The percentages of circulating lymphocytes are positively correlated with the percentages of splenic lymphocytes in normal rats. Interestingly, RIPC resulted in negative correlations between the percentages of splenic and circulating T lymphocytes, while the correlation between splenic and circulating B lymphocytes remained positive. For animals subjected to RIPC followed by MCAO, RIPC increased splenic volume with an expansion of splenic lymphocytes 3 days after MCAO. Furthermore, the removal of the spleen 1 day or 2 weeks before RIPC and MCAO reduced the protective effect of RIPC on ischemic brain injury and reversed the effects of RIPC on circulating immune cell composition. RIPC significantly reduced brain infiltration of Tc and NKT cells. Prior splenectomy showed no effect on immune cell infiltration after RIPC and stroke.

**Conclusion:**

These results reveal an immunomodulatory effect of the spleen, effecting mainly the spleen-derived lymphocytes, during RIPC-afforded neuroprotection against cerebral ischemia.

**Electronic supplementary material:**

The online version of this article (10.1186/s12974-018-1190-9) contains supplementary material, which is available to authorized users.

## Background

Stroke is the leading cause of lethality and permanent disability throughout the world. Accumulating evidence indicates that brief episodes of ischemic pre-treatment have a protective effect on subsequent cerebral ischemia-reperfusion injury [1–3]. Such ischemic preconditioning may vary greatly in terms of location, timing, and duration. The remote ischemic preconditioning (RIPC) of the limbs elicited tolerance against brain ischemia through brief blood flow constriction [4, 5]. Specifically, the noninvasive RIPC strategy of bilateral limb occlusion by tourniquet can be applied more readily to humans and has therefore become a focus for clinical translation [6–8]. RIPC has been shown to prevent the ischemic brain damage in patients during intracranial aneurysm treatment [9]. Individuals with peripheral vascular hypoperfusion, similar to RIPC, have significantly more favorable stroke outcomes [10]. Moreover, we have reported that brief repetitive bilateral upper arm ischemic preconditioning improves cerebral perfusion and reduces recurrent strokes in patients with intracranial arterial stenosis [7]. These clinical evidences suggest a neuroprotective effect of RIPC in stroke. However, the mechanism underlying RIPC-afforded neuroprotection is not clear.

The observation that RIPC in bilateral limbs can lead to brain protection draws our attention to the peripheral elements that function as the linkages between brain and hind limbs. Immune cells, particularly lymphocytes, have been verified to play an important role in the progression and prognosis after stroke [11–13]. Our previous study has shown that noninvasive RIPC changes the compositions of peripheral immune cells, ameliorates the post-middle cerebral artery occlusion (MCAO) reduction of T lymphocytes in the blood, and reverses the reduction of B cells [8]. These results suggest that changes in immune cells, especially the lymphocytes, in circulation may be a mechanism underlying the RIPC-mediated neuroprotection.

The spleen, the largest peripheral immune organ, is a reservoir for a variety of immune cells. Many studies have shown that during the acute phase of stroke, the spleen dramatically shrinks accompanied by the release of the immune cells into the circulation [14, 15]. The released immune cells subsequently infiltrate into the ischemic brain and contribute to brain injury [16, 17]. Observations from human studies identified that substantial post-stroke reduction in splenic volume occurred in approximately 40% of stroke patients. Some biological factors, including age, sex, race, and history of stroke, influenced post-stroke splenic contraction. Patients with splenic contraction showed higher percentages of blood lymphocytes and higher inflammatory cytokine levels in blood [14, 18]. Moreover, the post-stroke changes in splenic volume over time were biphasic, with an initial decrease followed by a delayed increase [18]. Such changes might correlate with the post-stroke inflammatory cascade. All these studies suggest an important role of spleen in stroke pathology. However, the effect of the spleen in RIPC-mediated neuroprotection after stroke has been unexplored.

In this study, we used surgical splenectomy to study the impact of the spleen on RIPC-mediated neuroprotection and peripheral lymphocyte changes after stroke. We discovered that RIPC resulted in dramatic changes in the compositions of immune cells (T lymphocyte subsets and B lymphocytes) in the spleen, which correlated with the lymphocyte changes in blood. Splenectomy impaired the protective effect of RIPC on ischemic brain injury and reversed the RIPC-induced peripheral lymphocyte changes, while showed no effect on immune cell infiltration into the ischemic brain. Taken together, our results reveal a novel immunomodulatory effect of the spleen underlying RIPC-afforded protection that warrants further mechanistic investigations.

## Methods

### Animals and grouping

Male Sprague-Dawley (SD) rats weighing 280–320 g (Vital River Laboratory Animal Technology Co. Ltd., Beijing, China) were used in this study. Animal care was carried out in accordance with guidelines approved by the Capital Medical University. All efforts were made to minimize any suffering and to reduce the number of animals used.

A total of 132 rats were used in this study. In order to detect the effect of RIPC on the spleen under the physiological status, 18 rats were randomly divided into three groups (*n* = 6 each group), from which spleens and blood were collected at different time points: pre-RIPC, RIPC-1 h, and RIPC-3 days. To clarify the role of RIPC on the spleen under the pathologic condition of stroke, 24 rats were divided into three groups: RIPC^−^MCAO^−^, RIPC^−^MCAO^+^, and RIPC^+^MCAO^+^ (*n* = 8 each group). At 3 days post reperfusion, the spleen weight and splenocytes were analyzed. In order to demonstrate the role of the spleen in the RIPC-mediated neuroprotection, 72 rats were randomly divided into three groups: MCAO, RIPC + MCAO, and splenectomy + RIPC + MCAO (*n* = 24 each group). Splenectomy was performed 1 day before RIPC, and RIPC was achieved 1 h before MCAO. Neurological deficits and motor behaviors were assessed over the 7 days after injury. Brain infarct, edema, and immune cell infiltration into the brain were measured 3 days post reperfusion. Moreover, the immune changes in circulation were detected by flow cytometry from the rats of RIPC + MCAO and splenectomy + RIPC + MCAO groups to explore the underlying mechanism. The other 18 rats were randomly divided into three groups: MCAO, RIPC + MCAO, and splenectomy + RIPC + MCAO (*n* = 6 each group), with the splenectomy performed 2 weeks before RIPC to limit the role of the body’s immune system responses due to a major surgery like a splenectomy.

### Splenectomy

Rats were anesthetized with 5% isoflurane inhalation (Lunan Pharmaceutical Group Corporation, Shandong, China) and maintained with 2% isoflurane inhalation (rodent ventilator model: ZS-M; ZS Dichuang Technology Co. Ltd., Beijing, China). A ~ 1-cm incision was made on the left side of the abdominal cavity under the rib cage. The spleen was removed by cutting the mesentery and connective tissue, and the splenic vessels were cauterized. For sham-control rats, incisions were made without removing the spleen.

### Remote ischemic preconditioning

Noninvasive remote ischemic preconditioning (RIPC) of the limbs was performed as described previously [6, 19]. Rats were anesthetized with 5% isoflurane inhalation and maintained with 2% isoflurane inhalation. Limb RIPC was achieved with four cycles of bilateral hindlimb ischemia (5 min/cycle, 40 min total). For each cycle, the proximal parts of the hindlimbs were tied with gauze ropes (13 cm × 13 cm) for 5 min, which was followed by 5 min of reperfusion with the gauze ropes untied. Non-RIPC animals were exposed to the same anesthesia, and untied gauze ropes were placed on both hindlimbs for 40 min. The preconditioning procedure was performed 1 h before middle cerebral artery occlusion (MCAO) or sham surgery.

### Transient focal cerebral ischemia and reperfusion

Immediately after RIPC, transient (90 min) focal cerebral ischemia was induced in male SD rats as previously described [20, 21]. Anesthesia was induced with 5% isoflurane and maintained with 2% isoflurane. Core body temperatures were maintained with a heating pad. The cerebral blood flow (CBF) during the surgery was measured by laser Doppler perfusion monitoring with a laser Doppler probe (PeriFlux System 5000, Perimed AB, Sweden) interfaced to a laptop equipped with the PeriSoft data acquisition software (Perimed Systems, Inc., Sweden) as previously described [22]. The CBF data of each animal were obtained at three time points (baseline, ischemia, and 10 min after reperfusion) and presented as the percentages of baseline. The animals with less than 60% reduction of cerebral blood flow or showing no obvious sign of neurological deficits (neurological score less than 2) after MCAO surgery were excluded. Exposure of the right MCA without occlusion was performed as sham surgery.

### Cerebral edema assessment

Brain water content (BWC) was quantified using the wet-dry method as previously described [23]. At 96 h post reperfusion, rats from each group were decapitated and the brains were rapidly removed. BWC was estimated in 3-mm coronal sections of the ipsilateral brain (or corresponding contralateral brain), centered upon the impact site. Tissue was immediately weighed (wet weight), then dehydrated at 65 °C. The sample was reweighed 48 h later to obtain a dry weight. The percentage of tissue water content was calculated using the following formula: BWC = [(wet weight) − (dry weight)/wet weight] × 100%.

### 2,3,5-Triphenyltetrazolium chloride staining

For 2,3,5-triphenyltetrazolium chloride (TTC) staining, the brains were removed rapidly on ice and sliced into seven coronal sections (2 mm thick). The sections were immersed in 2% TTC (Sigma-Aldrich, San Jose, CA) at 37 °C for 20 min and then fixed in 4% paraformaldehyde. Using the ImageJ 2× (National Institutes of Health, Bethesda, MD), the infarct size with edema correction was calculated as the area of the contralateral hemisphere minus the non-infarcted area of the ipsilateral hemisphere. Data were normalized to the non-ischemic brain and expressed as a percentage.

### Neurological deficit assessment

Neurological deficit assessment was performed by investigators blinded to the treatment groups. Rats were examined and assessed before surgery, 0.5, 24, 48, and 72 h after brain reperfusion. The Longa scoring system was used as follows: 0 = no deficit, 1 = failure to extend left forepaw, 2 = circling to the left, 3 = dumping to the left, 4= failure of spontaneous walking and loss of consciousness, and 5 = death.

### Balance beam test

The rats were placed on a narrow strip of wood (30 × 1.3 cm^2^). The scoring standards were as follows: 1 = four limbs were all on the wood in a balanced situation, 2 = limbs of one side were able to grasp the wood or shake on the wood, 3 = one or two limbs slipped from the wood, 4 = three limbs slipped from the wood, 5 = fell over after struggle on the wood, 6 = suspended on the wood and fell over after struggle, and 7 = fell over immediately without struggle at all [24].

### Foot fault test

The rats were placed on a net with 2.3 × 2.3 cm mesh size. When the rats were walking, the number of times the front paws fell through over 2 min was recorded. The calculation formula = (the number of the wrong steps of the left front paw − the number of the wrong steps of right front paw)/total steps.

### Leukocyte harvest from blood, spleen, and brain

Blood was taken from either the left or the right caudal vein with 1 ml syringe and rapidly transferred to heparin saline anticoagulant tubes. For each rat, 0.5–1 ml of venous blood was harvested. Spleen single-cell suspensions were obtained by grinding followed by filtration through a nylon mesh. For leukocyte retrieval, the samples from the blood and spleen were centrifuged at 3000 rpm for 4 min at 4 °C. The pellets were treated with the red blood cell lysis buffer (Beyotime Biotechnology Co. Ltd., Jiangsu, China) and washed twice with phosphate buffer solution (PBS, HyClone Laboratories Inc., Logan, UT) to remove red blood cells. For brain cell isolation, the contralateral and ipsilateral hemispheres were cut into small pieces, ground, and filtered through a 70-μm cell strainer (Miltenyi Biotec, Bergisch Gladbach, Germany) to obtain a single cell suspension. Cell pellets were resuspended in 70% Percoll (Solarbio, Beijing, China), transferred into 15 ml tubes, and then overlaid with 30% Percoll. The mononuclear cells were harvested after centrifuging at 2000 rpm for 25 min at room temperature [25]. The leukocytes were analyzed for membrane marker expression by flow cytometry.

### Flow cytometry analysis

Leukocyte classification by phenotypic analysis (the surface expression of antigen markers) was performed by flow cytometry. Leukocytes were resuspended in PBS at a concentration of 2 × 10^5^/ml and stained with the fluorochrome-conjugated antibodies in darkness for 30 min at room temperature. All antibodies were purchased from Biolegend (San Diego, CA), including fluorescein isothiocyanate (FITC) anti-rat CD3 (201403), peridinin chlorophyll protein (PerCP) anti-rat CD8a (201712), allophycocyanin (APC) anti-rat CD161 (205606), phycoerythrin (PE) anti-rat CD45RA (202307), and PE anti-rat CD4 (203307). Appropriate isotype-matched immunoglobulins were used as negative controls. An additional file shows this in more detail (please refer to Additional file 1: Fig. S1) Cells were analyzed on a FACSCalibur flow cytometer with Cell Quest software (Becton Dickinson, San Jose, CA, USA). The lymphocytes were gated on the scatter plots of forward scatter (FSC-H) and side scatter (SSC-H), excluding debris and cell aggregates; T cell subsets and B cells were further gated in the P1 population based on their expression of specific markers. We defined the CD3^+^CD4^+^ population as helper T (Th) cells, CD3^+^CD8^+^ population as cytotoxic T (Tc) cells, CD3^−^CD45RA^+^ population as B lymphocytes, and CD3^+^CD161a^+^ population as natural killer T (NKT) cells.

### Statistical analysis

The number of rats in each experimental group was determined by power analyses, informed by our past experience with similar measurements (*α* = 0.05 and *β* = 0.20). All data are expressed as mean ± SEM. Differences between two groups were evaluated for statistical significance using a Student’s *t* test. Differences between three or more groups were evaluated for statistical significance using one-way ANOVA followed by Bonferroni post hoc test. Normality of the distribution and homogeneity of variance were assessed by the *F* test or Bartlett’s test before the Student’s *t* test and the ANOVA, respectively. A two-tailed *p* value of < 0.05 was considered statistically significant. Correlation was analyzed with the Pearson correlation coefficient. The datasets used and/or analyzed during the current study are available from the corresponding author upon request.

## Results

### RIPC increased the number of lymphocytes in the spleen

**Fig. 1:**
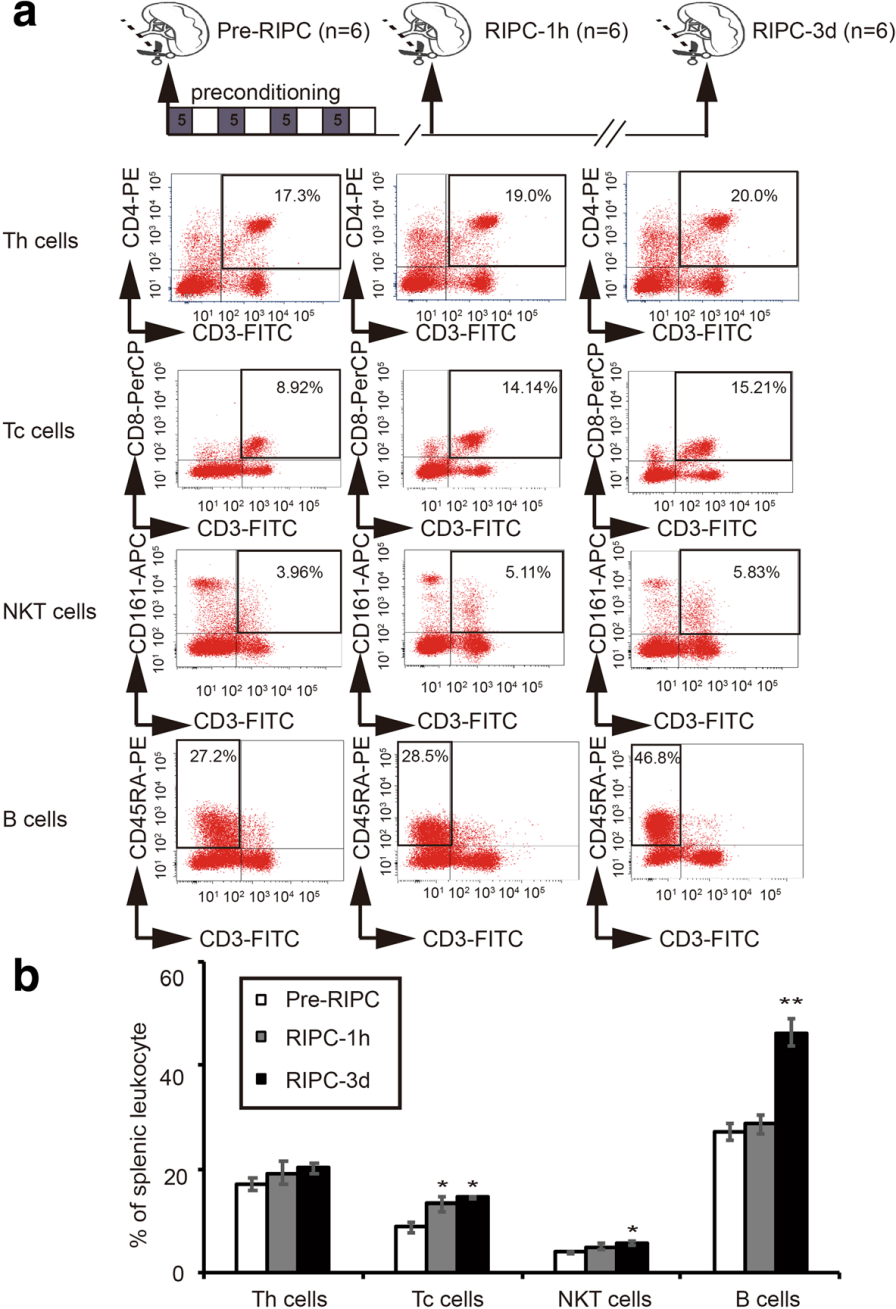
Limb remote ischemic preconditioning (RIPC) increases the splenic lymphocyte populations. RIPC was conducted by four cycles (10 min/cycle, 40 min total) of bilateral hindlimb ischemia. Spleens were removed before RIPC and 1 h and 3 days after RIPC. **a** Representative images of flow cytometry showing different splenic lymphocytes, including helper T (Th) cells (CD3^+^CD4^+^), cytotoxic T (Tc) cells (CD3^+^CD8^+^), natural killer T (NKT) (CD3^+^CD161^+^) cells, and B (CD3^−^D45RA^+^). Indicated numbers are the mean percentages of targeted cells. **b** Statistical analysis of splenic lymphocyte populations prior to and after RIPC. *n* = 6 rats per group. Data are presented as means ± SEM, *p < 0.05, **p < 0.01 versus corresponding lymphocytes before RIPC

In order to identify the effect of RIPC on the spleen, spleens were removed at different time points (before RIPC, 1 h and 3 days after RIPC) and splenic lymphocytes were analyzed by flow cytometry (Fig. 1a). RIPC increased the percentages of Tc cells as soon as 1 h after RIPC (Fig. 1b). The percentage of NKT cells was significantly increased 3 days after RIPC (Fig. 1b). Remarkably, RIPC dramatically increased the B cell percentage in the spleen 3 days after RIPC. These results suggest that RIPC can increase the percentages of splenic lymphocyte populations.

### The effect of RIPC on the correlation between splenic and peripheral lymphocytes

**Fig. 2:**
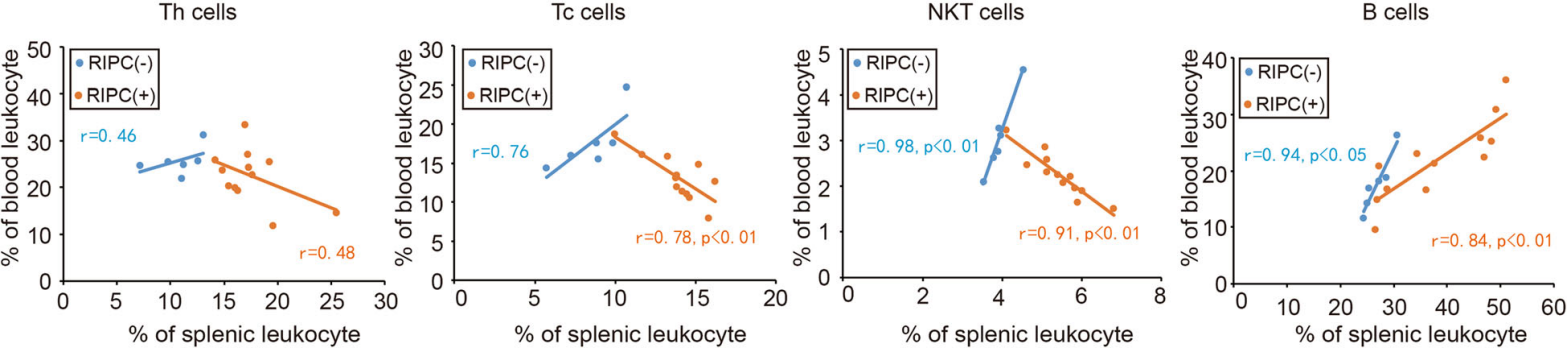
Correlation analyses between splenic and peripheral lymphocytes in rats with or without RIPC. Spleens and blood from caudal vein were collected from rats with or without RIPC. Different lymphocyte populations were detected by flow cytometry. Correlations between peripheral and splenic lymphocytes were analyzed. The data from rats without RIPC are presented in blue, and the data from rats with RIPC are presented in red

In our previous study, we found that RIPC alone decreased the percentage of T lymphocytes in peripheral blood after stroke, while the percentage of B cells in peripheral blood was slightly reduced in rats subjected to RIPC alone [8]. Spleen is a major reservoir of blood cells. We therefore analyzed the correlation between splenic and peripheral lymphocytes in order to detect the effect of RIPC on the communication between spleen and blood. As shown in Fig. 2, there is a positive correlation between splenic and peripheral lymphocytes in normal rats. RIPC induced a negative correlation between splenic and peripheral Th, Tc, and NKT cells, while the correlations between splenic and circulating B lymphocytes remained positive. These results suggest that RIPC may prevent the efflux of T lymphocytes into the circulation while enhancing B cell generation and/or release from spleen.

### RIPC increased splenic volume and lymphocyte number after MCAO

**Fig. 3:**
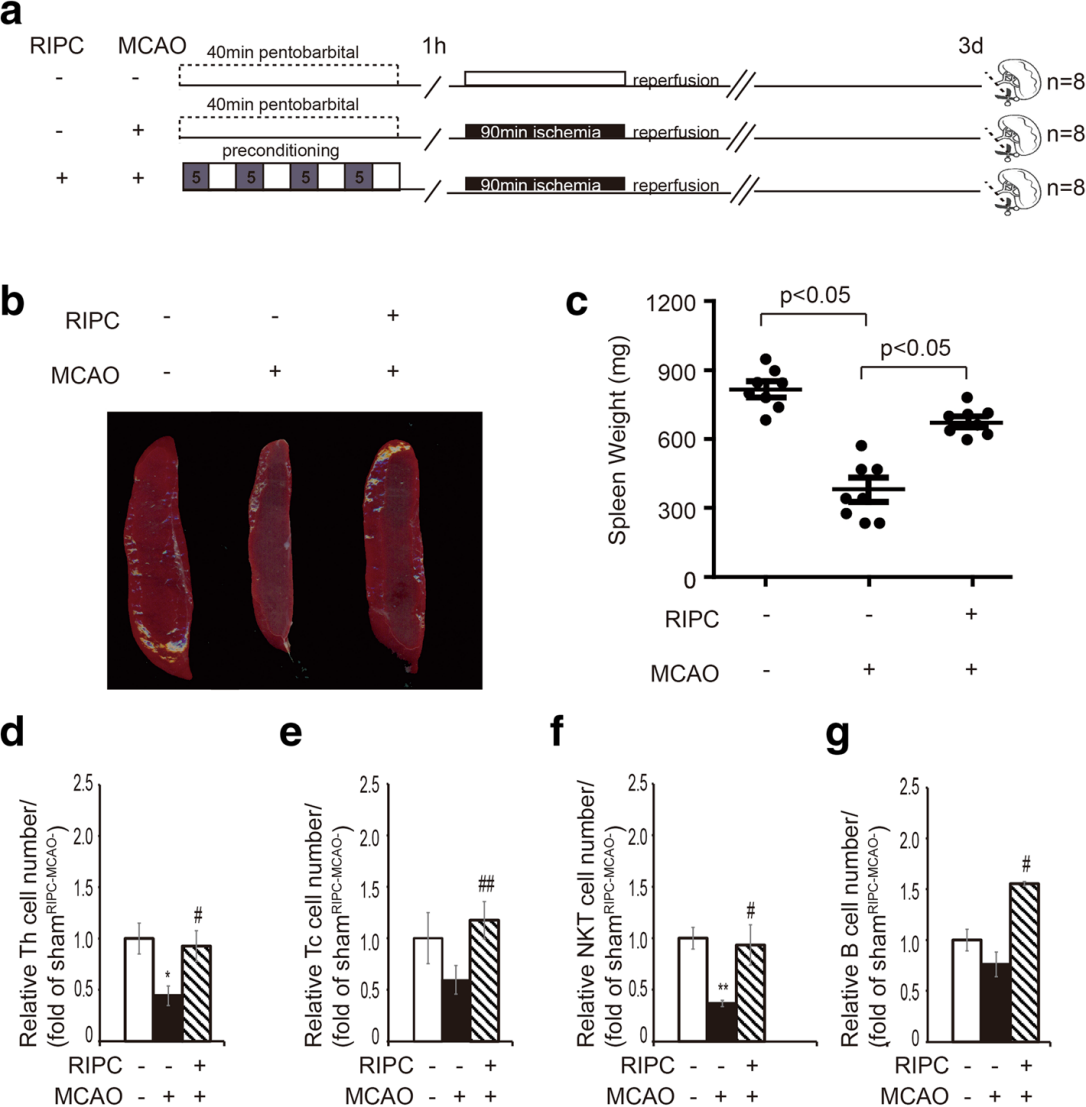
The effect of RIPC on splenic volume and lymphocyte populations followed by MCAO. **a** Experimental protocols. RIPC was conducted 1 h before MCAO. Non-RIPC animals were exposed to the same anesthesia for 40 min. Brain ischemia was induced by 90 min MCAO. Sham-operated animals underwent anesthesia and surgical exposure of the right middle cerebral artery without occlusion. Three groups of animals were prepared: RIPC^−^MCAO^−^, RIPC^−^MCAO^+^, and RIPC^+^MCAO^+^. **b** Representative spleen images from each group. **c** Spleen weight of rats in each group. **d**–**g** Quantification of the number of different splenic lymphocytes. Data are expressed as fold change of control group (RIPC^−^MCAO^−^). *n* = 8 rats per group. Data are presented as means ± SEM, *p < 0.05, **p < 0.01 versus RIPC^−^MCAO^−^ rats, ^#^*p* < 0.05, ^##^*p* < 0.01 versus RIPC^−^MCAO^+^ rats

In order to further clarify the effect of RIPC on the spleen under the pathological state of stroke, the spleens from MCAO rats with or without RIPC were collected 3 days after reperfusion (Fig. 3a). We found that the spleen shrank after MCAO (RIPC^−^MCAO^−^ vs. RIPC^−^MCAO^+^). However, RIPC could significantly maintain the volume and weight of the spleens (RIPC^−^MCAO^+^ vs. RIPC^+^MCAO^+^), as shown in Fig. 3b, c. Moreover, in rats without RIPC, the number of splenic lymphocytes decreased after MCAO, accompanying the reduced spleen volume. In contrast, the numbers of T cell subsets and B lymphocytes in the spleens significantly increased in MCAO rats which underwent RIPC (Fig. 3d–g). Together, these results suggest that RIPC can maintain the splenic volume and the number of splenic lymphocyte populations after stroke.

### Splenectomy reversed the protective effect of RIPC on ischemic brain injury

**Fig. 4:**
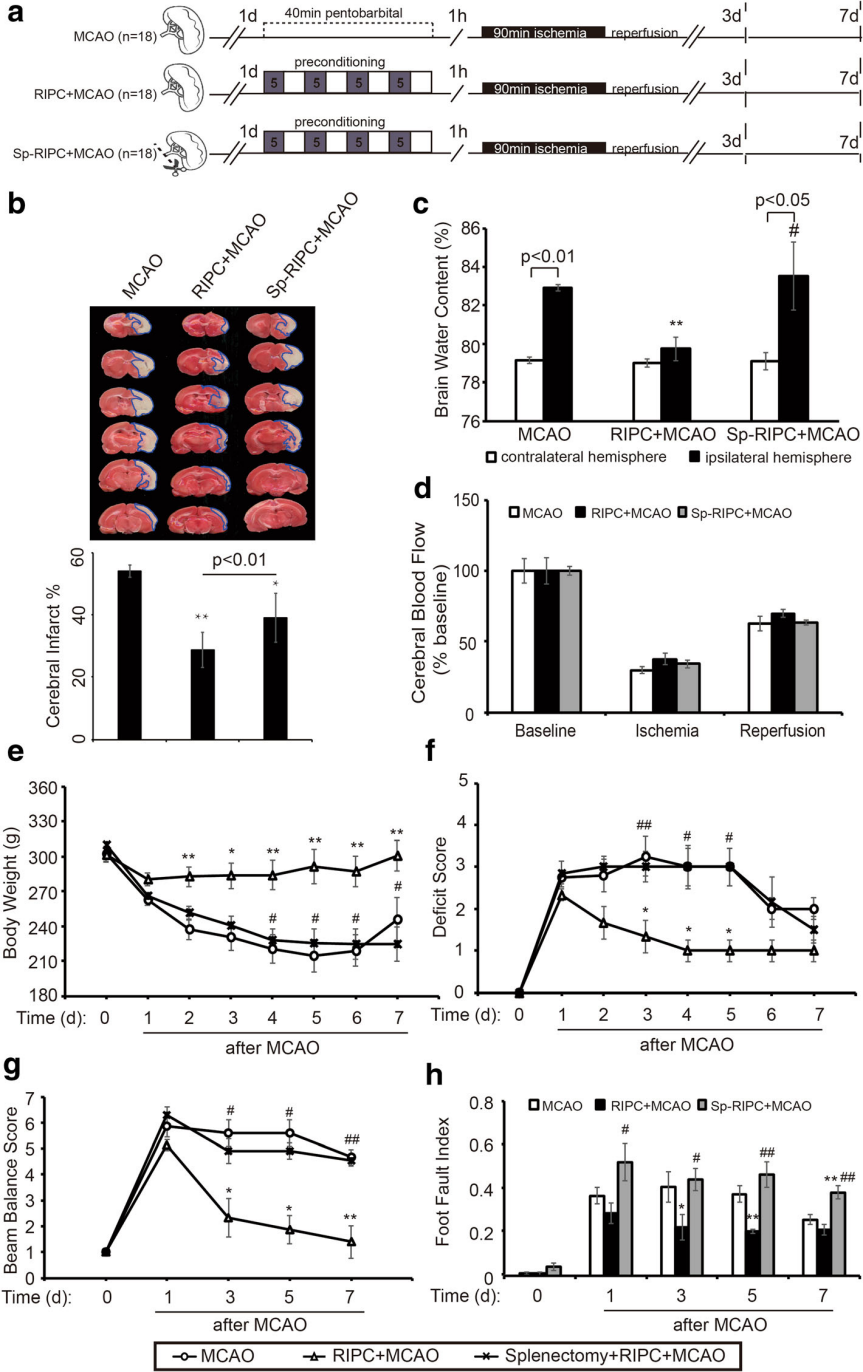
Splenectomy removes the protective effect of RIPC on ischemic brain injury. Splenectomy was conducted 1 day before 40 min RIPC and 90 min MCAO. Non-splenectomy animals underwent anesthesia and surgical exposure of the spleen without removal. **a** Experimental protocols. Three groups of animals were prepared: MCAO, RIPC + MCAO, and splenectomy + RIPC + MCAO (*n* = 18 per group). **b** Representative TTC images of animals from each group, *n* = 8 per group. The infarct areas are outlined with blue lines. **c** Brain water content of contralateral and ipsilateral hemispheres from each rat was detected through the wet-dry method. The calculating formula is BWC = [(wet weight) − (dry weight)/wet weight] × 100%, *n* = 4 per group. **d** Cerebral blood flow during the MCAO was measured at three time points: baseline, ischemia, and reperfusion. Data are normalized to baseline and expressed as percentages. **e** Body weight of all rats from each group was measured over the 7 days after MCAO. **f**–**h** Neurological function and sensorimotor behaviors were assessed by neurological deficit score, beam balance score, and foot fault index, *n* = 6 rats per group. Data are presented as means ± SEM, *p < 0.05, **p < 0.01 versus MCAO group. ^#^*p* < 0.05, ^##^*p* < 0.01 versus RIPC + MCAO rats

Rats subjected to RIPC demonstrated significant reduction in infarct volumes (Fig. 4a, b) and cerebral edema (Fig. 4c) compared to animals without RIPC 3 days after MCAO. Pre-stroke splenectomy attenuated the RIPC-induced reduction in brain infarct sizes and edema (Fig. 4b, c). Meanwhile, cerebral blood flow, detected during the MCAO surgery, was reduced to less than 40% of baseline during ischemia and reestablished to 70% of baseline after reperfusion without significant differences between groups (Fig. 4d). Moreover, consistent with the increased brain injury, rats subjected to splenectomy + RIPC exhibited decreased body weight (Fig. 4e) and impaired neurological function and sensorimotor behavior, as manifested by the neurological deficit score, beam balance score, and foot fault index, lasting at least through 7 days post reperfusion (Fig. 4f–h).

**Fig. 5:**
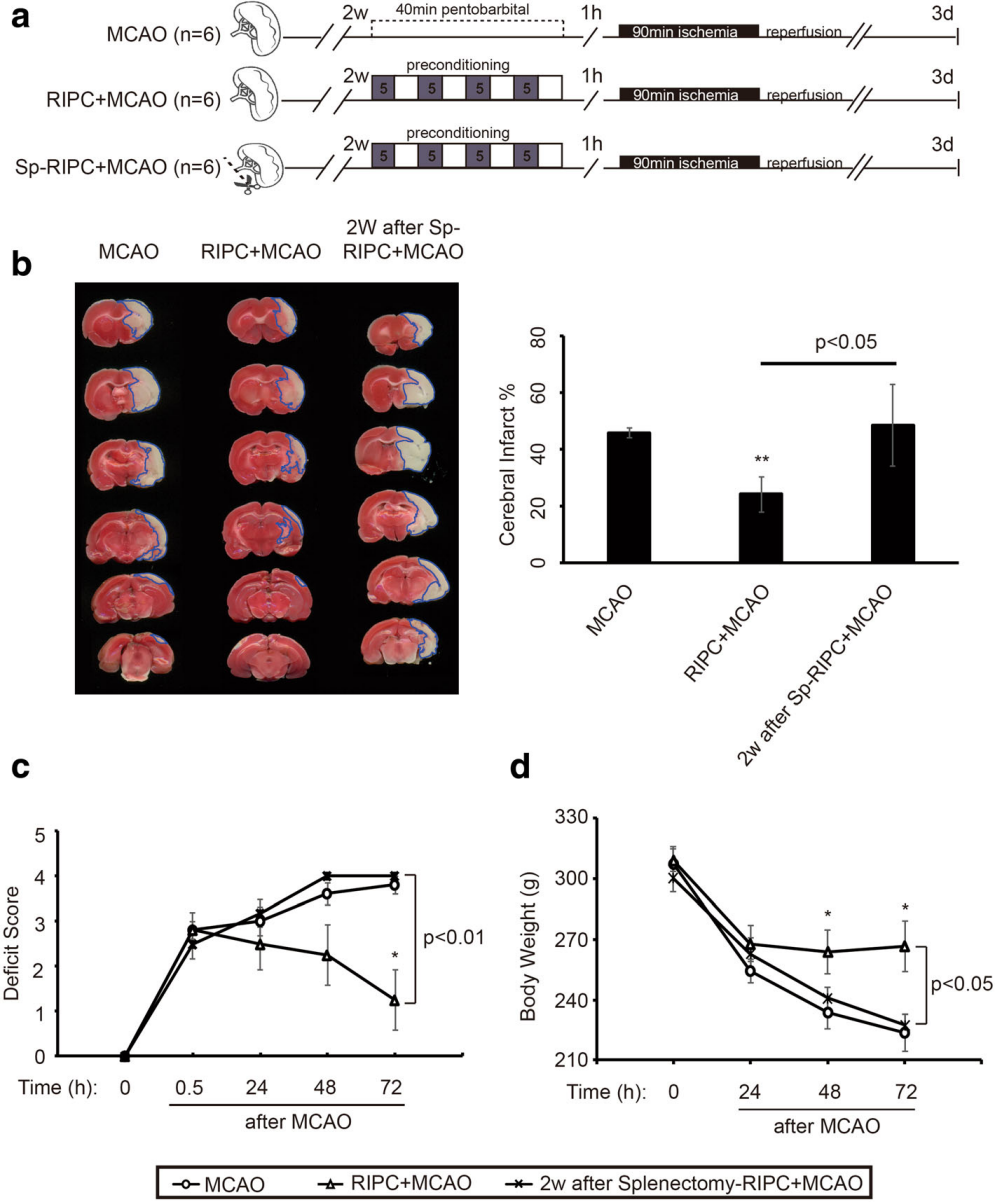
Splenectomy 2 weeks before RIPC and MCAO also removed the protective effect of RIPC on ischemic brain injury. Splenectomy was conducted 2 weeks before 40 min RIPC and 90 min MCAO. Non-splenectomy animals underwent anesthesia and surgical exposure of the spleen without removal. **a** Experimental protocols. Three groups of animals were prepared: MCAO, RIPC + MCAO, and splenectomy + RIPC + MCAO (*n* = 6 each group). **b** Representative TTC images of animals from each group. The infarct areas are outlined with blue lines. **c** Neurological deficit assessment was performed with the Longa scoring system during the 3 days after MCAO. **d** Body weight of all rats from each group were measured during the 3 days after MCAO. Data are presented as means ± SEM, *p < 0.05, **p < 0.01 versus MCAO group. ^#^*p* < 0.05, ^##^*p* < 0.01 versus RIPC + MCAO rats

The body’s immune system is still responding to splenectomy when MCAO was performed 1 day later, possibly causing immune cell fluctuations and confounding the data on the effects of splenectomy on RIPC and stroke. Therefore, splenectomy was performed 2 weeks prior to RIPC and MCAO to confirm the role of the spleen in RIPC-afforded neuroprotection. Splenectomy 2 weeks before RIPC and MCAO fully removed the protective effect of RIPC on ischemic brain injury (Fig. 5). Taken together, these results demonstrate that splenectomy removed the protective effect of RIPC on ischemic brain injury, suggesting an important role for the spleen in RIPC-mediated neuroprotection against stroke.

### Splenectomy removed RIPC-induced changes in peripheral lymphocyte population after MCAO

**Fig. 6:**
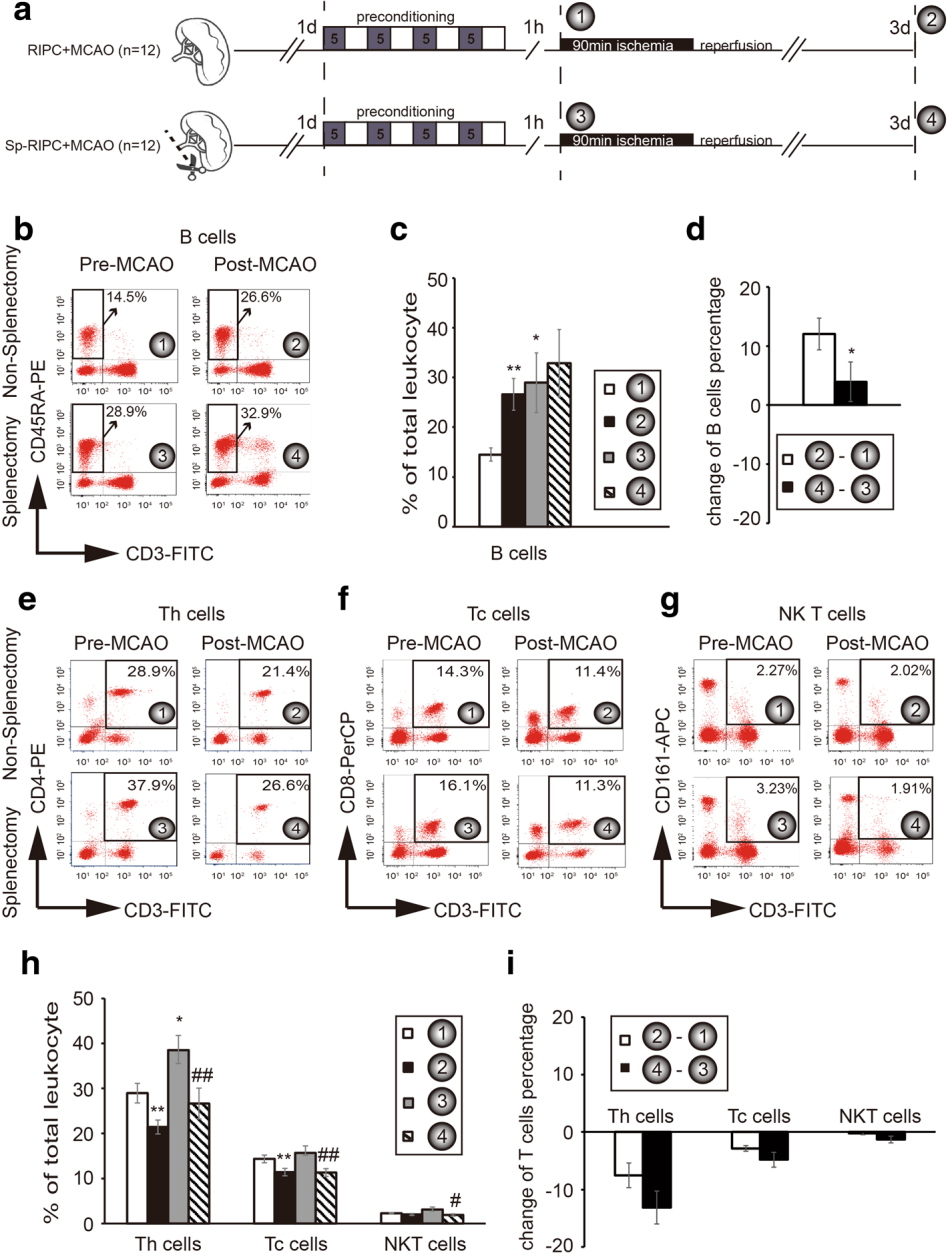
The effect of splenectomy on peripheral lymphocytes before RIPC and MCAO. **a** Experimental protocols. Two groups of animals were adopted: non-splenectomy + RIPC + MCAO and splenectomy + RIPC + MCAO. Blood was collected from the caudal tail vein at 1 h after preconditioning (① and ③) and 3 days after brain reperfusion (② and ④). **b**–**d** Flow cytometry analysis of B lymphocytes. **e**–**i** Flow cytometry analysis of T lymphocyte subsets. ②-① reflects the change of lymphocyte populations prior to and after RIPC + MCAO without splenectomy; ④-③ reflects the change of lymphocyte populations prior to and after MCAO with splenectomy and RIPC. Data are expressed as means ± SEM for 12 independent experiments. *p < 0.05, **p < 0.01 versus ① or ②-①; ^#^*p* < 0.05, ^##^*p* < 0.01 versus ③

Our previous studies revealed dramatic immune changes in peripheral blood composition after RIPC neuroprotection against cerebral ischemia [8]. Here, we explored the effect of splenectomy on RIPC-induced changes in circulating immune populations after stroke (Fig. 6a). As shown in Fig. 6b, c, RIPC robustly elevated the percentage of peripheral B cells in animals without splenectomy after MCAO (① vs. ②). Splenectomy alone significantly elevated the blood B cell population after RIPC (① vs. ③), whereas levels of B cells in animals with splenectomy remained unchanged after MCAO (③ vs. ④). The increase of blood B cell composition was maximized after splenectomy + RIPC so there is no further increase after MCAO (②-① vs. ④-③ in Fig. 6d).

Splenectomy increased the blood composition of Th cells after RIPC (① vs. ③ in Fig. 6e), and RIPC significantly decreased the peripheral T lymphocytes both in animals without or with splenectomy (① vs. ② and ③ vs. ④ in Fig. 6e–h). Splenectomy showed no significant effect on RIPC-induced T lymphocyte changes in stroke rats (Fig. 6i).

### The effect of RIPC and splenectomy on immune cell infiltration into the ischemic brain

**Fig. 7:**
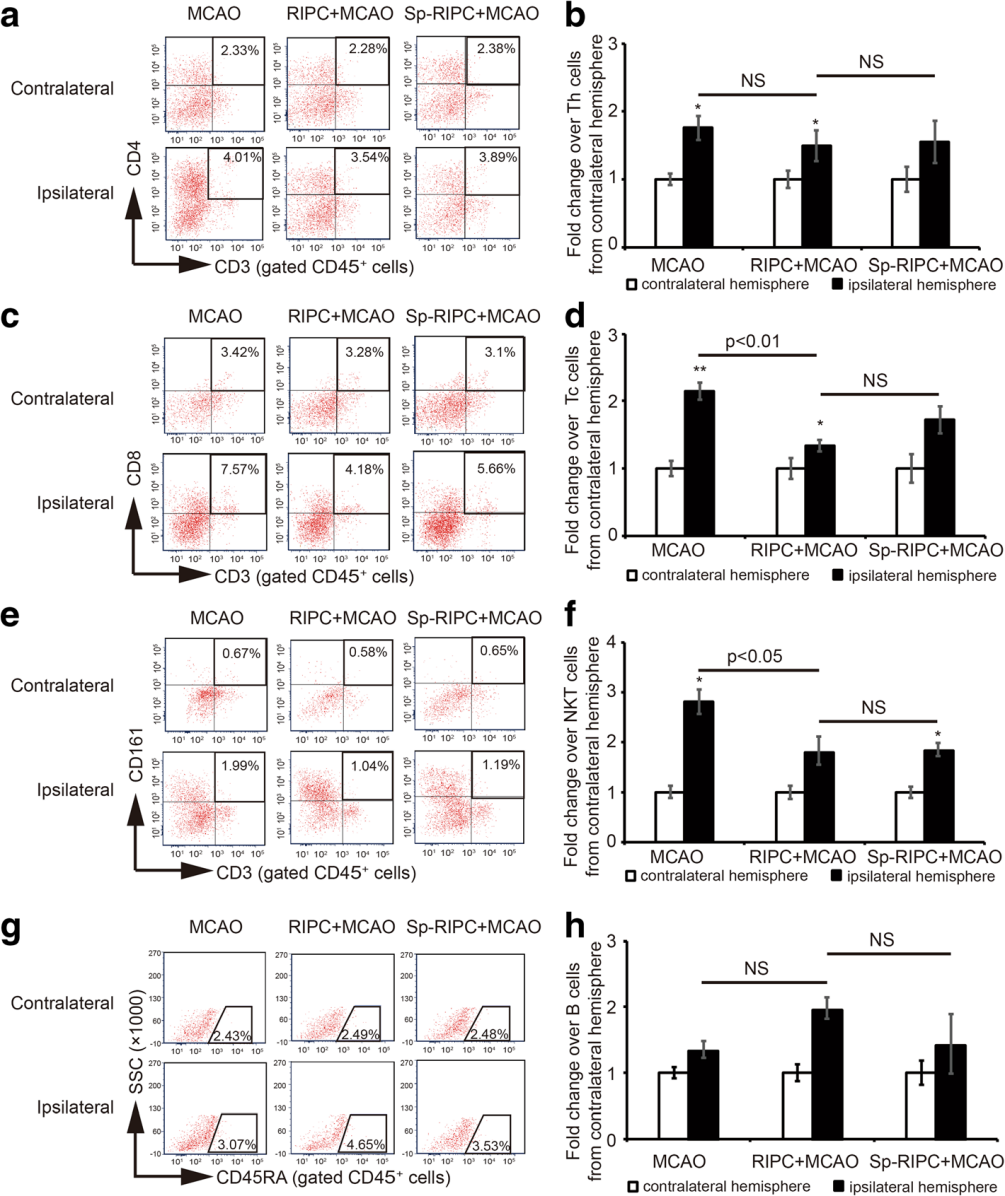
The effect of RIPC and splenectomy on immune cell infiltration into the ischemic brains. Three groups of animals were prepared: MCAO, RIPC + MCAO, and Sp-RIPC + MCAO (*n* = 6 per group). Splenectomy was conducted 1 day before 40 min RIPC and 90 min MCAO. Non-splenectomy animals underwent anesthesia and surgical exposure of the spleen without removal. The brains were collected at 3 days after MCAO. **a**, **c**, **e**, **g** Representative plots of FACS showing Th, Tc, NKT, and B cells in contralateral and ipsilateral hemispheres. Indicated numbers are the mean percentages of targeted cells. **b**, **d**, **f**, **h** Statistical analysis of different lymphocyte populations in contralateral and ipsilateral hemispheres. Data are normalized to the mean of respective contralateral brains and presented as means ± SEM, *p < 0.05, **p < 0.01 versus corresponding lymphocytes in contralateral hemispheres

MCAO significantly increased the infiltration of Tc and NKT cells in the ipsilateral hemispheres, which was inhibited by RIPC (Fig. 7c–f). Splenectomy prior to RIPC showed no significant effect on T lymphocyte infiltration into the ischemic brains as compared to RIPC alone group. RIPC or splenectomy had minimal effect on Th cell infiltration after stroke (Fig. 7a, b). There was no obvious infiltration of B cells into the brain 3 days after stroke (Fig. 7g, h). Although RIPC and splenectomy induced a robust elevation of peripheral B cell population (Fig. 6b, c), they had no significant effect in B lymphocyte infiltration 3 days after MCAO (Fig. 7g, h).

## Discussion

Ischemic stroke results in a rapid systemic inflammatory response, which exacerbates the initial infarct [26, 27]. Modulation of immune and inflammatory responses represents an important target for improving clinical outcomes after stroke [28]. Our previous study indicated that RIPC dramatically altered the levels of multiple immune cell populations and cytokines in blood [8]. However, how RIPC induced these alterations in the peripheral immune system has remained elusive.

Over the past decade, the contribution of the spleen to ischemic brain damage has gained considerable attention in stroke research. There is increasing evidence that the spleen-mediated peripheral immune changes contribute to progressive injury in a number of acute neurological disorders, including spinal cord injury, hemorrhagic stroke, and traumatic brain injury [29–32]. A recent study demonstrated that multipotent adult progenitor cells enhance recovery after stroke by modulating the splenic immune response [33]. However, whether RIPC modulates the splenic response is not clear. In this study, we discovered that in normal subjects, RIPC increased the splenic lymphocyte populations; meanwhile, RIPC decreased the T lymphocytes and robustly elevated the percentage of B cells in peripheral blood (Figs. 1 and 2). Under the pathologic condition of stroke, RIPC also maintained the splenic volume and lymphocyte numbers (Fig. 3). Moreover, removal of the spleen 1 day or 2 weeks before RIPC deprived RIPC-afforded neuroprotection 3 days post reperfusion, which lasted through day 7 after stroke (Figs. 4 and 5). Splenectomy also reversed RIPC-induced peripheral lymphocyte changes (Fig. 6). These results suggest that immunomodulation of the splenic response by RIPC may create a special immune environment that influences the progression of ischemic stroke.

We have shown previously that RIPC could reduce brain infarct early after stroke [8]. Splenectomy 2 weeks before stroke has been shown to be neuroprotective [34–36], while splenectomy shortly before stroke showed minimal protection [37]. Interestingly, in our study, splenectomy performed either 1 day or 2 weeks before RIPC and MCAO reduced and eliminated RIPC-afforded neuroprotection, respectively. Splenectomy shortly before MCAO may initiate an acute activation of the immune response, leading to tissue damage that cancels its protective effects in the brain [38]. This is supported by the fact that peripheral lymphocytes increased in the blood after splenectomy. Such immune cell elevation might be due to the mobilization of immune cells from other organs or tissues. For example, bone marrow is the primary site of hematopoiesis. All types of myeloid and lymphoid lineages are created in the bone marrow. Under normal conditions, lymphoid cells must migrate to secondary lymph organs, such as the spleen and lymph nodes, to complete maturation. However, when the spleen was removed, the role of the bone marrow as a peripheral immune cell source should not be ignored. Interestingly, our data further showed that splenectomy 2 weeks before RIPC and MCAO fully removed the protective effect of RIPC on ischemic brain injury (Fig. 5), suggesting that the spleen may contribute to RIPC-induced protection against CNS injury through complicated mechanisms of immune modulation over time.

Lymphocytes, especially T lymphocytes, play a destructive role after stroke [39, 40]. In our study, we found that exposure to RIPC could dramatically reduce the number of T cells especially CD8^+^ T cells and NKT cells in the blood prior to stroke. Interestingly, we found an increase of splenic T cells after RIPC in this study. Correlation analysis indicates that the splenic lymphocytes were proportional to the blood lymphocytes under normal physiological conditions, which is consistent with the public knowledge that circulating immune cells continuously migrate into and out of the resting spleen [41]. However, RIPC reversed the positive correlation between splenic and peripheral T lymphocytes, suggesting that the RIPC-induced decrease of T lymphocytes in the periphery might be due to the retention of circulating cells in the spleen and impaired outflow of splenic lymphocytes into the blood. It is unclear from the current study how RIPC increases splenic T or B lymphocytes so robustly after RIPC. We know that CD4 and CD8 T cells are mobilized from the spleen and redistributed to the non-lymphoid tissues following recognition of cognate antigens in the splenic white pulp [41]. Existing literature shows that bacteria/virus infection can induce recruitment of monocytes, NK cells, and neutrophils to the spleen, which is important for the infection prognosis through downstream adaptive immunity regulation [42–45]. However, whether RIPC can also induce recruitment of immune cells to the spleen or inhibit the egress of spleen immune cells into the circulation warrants further exploration. Moreover, in our previous research, we found a dramatic reduction in the percentage of circulating T cell subsets (including CD4^+^ T cell, CD8^+^ T cell, and NKT cells) after stroke, which was ameliorated by RIPC after stroke [8]. We found here that RIPC reduced the infiltration of Tc and NKT cells into the ischemic brain, which is consistent with our hypothesis that RIPC reduced the influx of immune cells after stroke. Splenectomy showed minimal effects on RIPC-induced reduction of T cells in blood or infiltration into the ischemic brain after stroke, suggesting that splenectomy removed the neuroprotective effect of RIPC through mechanisms probably independent of immune cell infiltration after stroke.

In our study, B cells show the most dramatic changes after RIPC. Our previous data revealed an elevation of circulating B cells in RIPC animals, which may be essential for RIPC-afforded protection against ischemia [8]. In this study, there is also an intriguing increase of splenic B lymphocytes at 3 days after RIPC. Correlation analysis shows that the B lymphocytes are elevated in the spleen and periphery at the same time even after RIPC, indicating that an upregulation of B cells in the blood might be due to increased B cell generation and release from the spleen. It is noted that splenectomy also mediated a high percentage of B cells in the blood. However, these non-splenic B cells might come from different sources, such as the bone marrow or lymph nodes, and possess functions distinct from B cells in the spleen. RIPC and splenectomy showed minimal effect on B cell infiltration. Different from the detrimental role of T lymphocytes in ischemic brain injury, recent findings demonstrate that B lymphocytes play a heterogeneous role in the adaptive immune response to stroke [12]. On the one hand, many studies show a special population of regulatory B lymphocytes secrete IL-10 and are protective in cerebral ischemia/reperfusion injury [46, 47]. A recent study reported that repetitive hypoxic preconditioning induces an immunosuppressive B cell phenotype prior to stroke onset, thereby preventing the infiltration of other immune cells into the brain and protecting the brain against ischemic attack [48]. On the other hand, B cell and antibody accumulation in the infarct region correlated with impairments in hippocampal long-term potentiation, resulting in short-term memory deficit weeks after stroke [49], which suggests the post-stroke detrimental effects of B cells. It is therefore warranted to explore whether the robustly increased peripheral B lymphocytes after RIPC or splenectomy possessed distinct functions and played critical roles in stroke outcomes.

## Conclusion

Our study suggests that the spleen plays a pivotal role in RIPC-mediated alterations in the peripheral immune system and following neuroprotection. Immunomodulation of the splenic response by noninvasive remote ischemic preconditioning may create a favorable systemic immune milieu to protect against ischemic brain injury and could serve as a platform to develop therapeutic approaches for stroke as well as other acute CNS injuries in the future.

## Additional file

Additional file 1: The flow cytometry scatter plots for isotype controls that were used as negative control. (PDF 120 kb)

## Abbreviations

APC: Allophycocyanin
BWC: Brain water content
CBF: Cerebral blood flow
CNS: Central nervous system
FITC: Fluorescein isothiocyanate
FSC: Forward scatter
MCAO: Middle cerebral artery occlusion
PE: Phycoerythrin
PerCP: Peridinin chlorophyll protein
RIPC: Remote ischemic preconditioning
SD: Sprague-Dawley
Sp: Splenectomy
SSC: Side scatter
Tc: Cytotoxic T
Th: Helper T
TTC: 2,3,5-Triphenyltetrazolium chloride
