# Supplementary figures and images for: Splenic responses play an important role in remote ischemic preconditioning-mediated neuroprotection against stroke
Source: J Neuroinflammation. 2018 May 28;15:167. doi: 10.1186/s12974-018-1190-9 (PMC5972448; doi:10.1186/s12974-018-1190-9)

Negative Control of:

Th cells

Tc cells

NKT cells

B cells

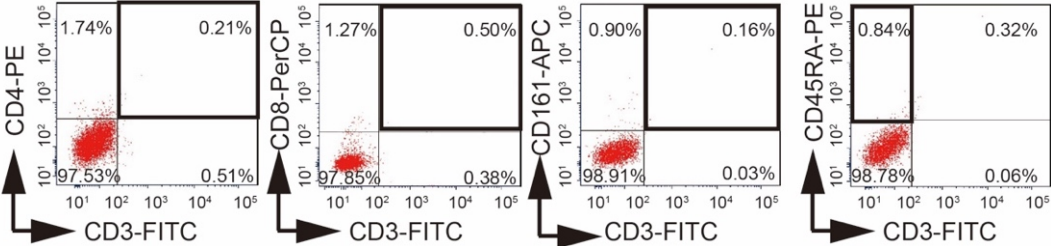

Additional file 1

Supplement: Supplementary file 1 — The flow cytometry scatter plots for isotype controls that were used as negative control. (PDF 120 kb) [file 12974_2018_1190_MOESM1_ESM.pdf]
